# For when you just can’t talk to ‘normal’ people . . . Exploring the use of informal support structures by supernumerary university paramedic students: findings from a phenomenological study

**DOI:** 10.29045/14784726.2023.3.7.4.1

**Published:** 2023-03-01

**Authors:** Mark Garratt

**Affiliations:** Coventry University (retired); Staffordshire University (student) ORCID iD: https://orcid.org/0000-0001-6266-2825

**Keywords:** coping strategies, paramedic students, peer support

## Abstract

**Background:**

In an effort to shield them from distress, pre-hospital ambulance staff may avoid discussing traumatic workplace experiences with friends and family. As a source of informal support, however, workplace camaraderie is considered important for managing occupational stress. For supernumerary university paramedic students there is limited research concerning how such experiences are managed, and whether they may benefit from similar informal support. This is a concerning deficit when contextualised by reports of higher stress levels among students undertaking work-based learning, and among paramedics / paramedic students in general. These original findings allude to how university paramedic students who are supernumerary within the pre-hospital workplace utilise informal support mechanisms.

**Methods:**

A qualitative, interpretive approach was adopted. University paramedic students were recruited via purposive sampling. Audio-recorded face-to-face semi-structured interviews were performed and transcribed verbatim. Analysis involved initial descriptive coding and then inferential pattern coding. The identification of themes and discussion topics was facilitated by a review of the literature.

**Results:**

Twelve participants were recruited aged from 19 to 27 years, and 58% (n = 7) were female. While most participants cited that they were able to enjoy the informal stress-relieving camaraderie of ambulance staff, there were perceptions that supernumerary status may leave them potentially isolated within the workplace. Participants may also compartmentalise their experiences away from friends and family in a manner similar to that found among ambulance staff. Informal student peer support networks were praised as a source of information and for emotional support. Self-organised online chat groups were ubiquitous as a means of keeping in touch with student peers.

**Conclusions:**

While undertaking pre-hospital practice placements, supernumerary university paramedic students may not have complete access to the informal support of ambulance staff, and they may feel unable to discuss stressful feelings with friends or loved ones. However, within this study, self-moderated online chat groups were used almost universally as a readily accessible means of peer support. Paramedic educators ideally need an awareness of how such groups are used to ensure that they represent a supportive and inclusive space for students. Further research into how university paramedic students utilise online chat groups for peer support may further reveal a potentially valuable informal support structure.

## Introduction

The mental health of emergency ambulance staff is currently of great concern. National surveys suggest that a high proportion of emergency ambulance staff suffer from poor mental health (including symptoms of depression, anxiety and post-traumatic stress disorder) and that male paramedics within the United Kingdom have a suicide rate 75% higher than the national average (Mind, 2019; Office for National Statistics, 2017). Additionally, in *Blue light: Post coronavirus research findings*, Mind (2021) cites that 77% of ambulance respondents felt that their mental health had worsened during the pandemic, with 32% now considering their mental health to be ‘poor’ or ‘very poor’.

As an informal and readily available support structure, being able to discuss worries and share experiences with like-minded peers is considered to represent a key interpersonal resource among paramedics. Chatting, joking and decompressing with crewmates after a traumatic call are cited to be the most commonly used methods of managing occupational stress, with some individuals displaying exacerbated symptoms when isolated from such contact (Clompus & Albarran, 2016; Mildenhall, 2012). As a source of emotional resilience, informal peer support may have even greater significance for pre-hospital care workers, as individuals have been found to avoid sharing emotive or traumatic experiences with family and non-work friends (Clompus & Albarran, 2016). Shakespeare-Finch et al. (2002) further suggest that some ambulance staff compartmentalise their experiences from loved ones and hypothesise that this may be due to having a repertoire of prior experience which acts as an emotional buffer.

Due to their lack of exposure, university paramedic students undertaking supernumerary pre-hospital placements are unlikely to have a repertoire of experience to fall back on. Should they feel the same imperative to keep their personal lives separate, they would also logically be denied the potential support of family and friends. In addition, their ability to engage with informal support may be complicated by the perforce peripheral nature of their participation as learners within the professional community, something which may be exacerbated by their supernumerary status (Hyde & Brady, 2002; Joyce, 1999).

While there is some evidence within broader healthcare highlighting the potential value of peer support between students (Green, 2018), there is an acknowledged dearth of literature which explores the experience and management of stress among university paramedic students (Warren-James et al., 2021). This is a concerning deficit when contextualised by findings of higher generic stress levels among university students who undertake work-based learning, and suggestions that both paramedics and student paramedics suffer from higher rates of occupational stress than the general population (Deasy et al., 2014; Hichisson & Corkery, 2020; Stallman, 2010). These findings offer some insight into how supernumerary university paramedic students utilise informal support structures.

## Methods

### Paradigm of enquiry and reflexivity

The overarching study was a subjective enquiry which sought to explore the workplace enculturation and professional identity development of supernumerary university paramedic students. An interpretive approach aligned with the work of seminal phenomenologist Martin Heidegger (1927) was adopted as a means of providing analytical structure (see Supplementary 1). In alignment with this interpretive approach, a qualitative methodology involving individual semi-structured interviews with pre-formulated questions was adopted.

Finlay (2008) suggests that the researcher’s personal bias and pre-conceptions should be acknowledged and utilised to appreciate the meaning that individuals attribute to experience, an approach considered to be unique to phenomenological research (Paley, 2017). At the time of the study, the author had been a qualified paramedic for over 20 years and a university paramedic educator for 12 years. In alignment with Heidegger’s (1927) hermeneutic ethos, the author’s own ‘position’ and potential for interpretative bias was acknowledged throughout the study.

### Sampling strategy and sample size

The target population was full-time university paramedic students who were supernumerary in clinical practice. For ease of access, only students at the author’s employing university were included. A face-to-face invitation and explanation of the study was given to all students, and expressions of interest were requested via email or in person. As the focus was upon acquiring in-depth information from specific individuals, purposive sampling allowed for a small sample to be balanced with the deliberate selection of participants. The selection was intended to represent diversity in terms of sex, ethnicity, age and route of entry to university. Age and life experience are considered to play a role in pre-hospital enculturation (Lazarsfeld-Jenson et al., 2011), while route of entry to university may offer an indication of socioeconomic status (Wyness, 2017). Participants were also selected from a mix of cohorts so as to compare their levels of workplace exposure (Table 1).

**Table 1.** Participant attributes.

	Participant attributes							
**Participant interview order**	Female	Male	Age > 21	Age ≥ 21	Asian heritage	Year 1 FdSc	Year 2 FdSc	New BSc cohort
**P1**		/	/				/	
**P2**		/		/			/	
**P3**	/		/			/		
**P4**	/			/			/	
**P5**		/		/	/		/	
**P6**	/			/			/	
**P7**	/			/			/	
**P8**		/		/		/		
**P9**	/		/				/	
**P10**		/	/				/	
**P11**	/			/	/	/		/
**P12**	/			/		/		/

BSc: Bachelor of Science; FdSc: foundation degree.

From a total of 20 volunteers, 10 participants were selected. However, as data collection progressed it became apparent that participants had some experiential ‘distance’ from being ‘brand new’ to the pre-hospital workplace. Volunteers were therefore requested from a new BSc cohort who were still within their initial period of placement. Two more participants were included.

### Data collection methods

Each participant had one 60-minute face-to-face semi-structured interview in a pre-booked room. In accordance with Cohen et al. (2010), this facilitated familiarity and privacy, enabled prior arrangement of the environment and minimised potentially harmful interruptions. Prior to their interview, it was confirmed that each participant understood the nature of the study, and their voluntary informed consent was requested in accordance with the British Educational Research Association (BERA) (2018). All interviews were audiotaped and transcribed verbatim by the author. In accordance with BERA (2018), recordings and transcriptions were stored on a password-protected encrypted computer on secure premises.

### Data analysis

Completed transcripts were not validity checked by interviewees, in accordance with Basit’s (2010) assertion that when seeing their words in isolation participants may wish to change something which seems embarrassing or judgemental. Each transcript was entered into the software package NVivo12, which facilitated coding and tagging of data. It additionally allowed for the storage of reflexive memos, and for links to be created to the transcript. Data were coded through applying initial descriptive category codes, and then more inferential pattern codes (Miles & Huberman, 1994).

In accordance with Paley’s (2017, p. 148) assertion that a ‘background theory’ is essential for meaningful phenomenological analysis, the overarching research incorporated Heidegger’s (1927) conception of ‘Dasein’. This relates to how individuals view themselves within social contexts and is underpinned by the temporal arrangement of ‘care’ (see Supplementary 1). This suggests that everyday interactions are embodied by an individual negotiation of past experience (‘thrownness’), present concerns (‘fallenness’) and future aspirations (‘projection’) (Heidegger, 1927). This framework provided the superordinate a priori themes, within which subordinate themes were located as they arose (see Supplementary 1). Analysis was undertaken solely by the author as part of a doctoral thesis.

### Data saturation

The decision to cease data collection after 12 interviews was based upon the practicalities of analysing the volume of collected data; the diminishing number of new codes which were emerging; and the perception that there were enough original data to meet the objectives. This accords with the ‘diminishing returns’ stance to data saturation taken by Mason (2010, p. 16).

## Results

Four areas were identified which relate to the use of informal support for university paramedic students:

- benefitting from the informal support of ambulance staff;
- the potential barrier of supernumerary status;
- benefitting from the informal support of student peers; and
- the ubiquitous nature of online chat groups.

### Benefitting from the informal support of ambulance staff

Participants described the benefits that they felt in being included with the informal conversations and humour used by ambulance staff after attending a stressful incident. This was broadly conveyed by two participants:

It’s a stressful job. You might go to some horrible job, and you just want to have a laugh afterwards, ‘cause you feel otherwise you’ll cry. And you just wanna be like able to relax, chat about anything you want . . . just chill out and re-align your thoughts on what you’re there for. (Participant 3)

It’s like quite stressful, but ’cos everyone was so light-hearted all the time, you close the door on like a bad job and you’d be joking straight away, it helped a lot – like you don’t have to sit and dwell on it . . . (Participant 6)

Participant 8 conveyed a clear desire to be with his mentor and around other ambulance staff after attending a traumatic road traffic collision. In this case he had travelled home to replace contaminated uniform and returned to complete the shift. For this participant, the desire to interact with ambulance staff was juxtaposed by an unwillingness to burden his mother:

I think my mentor was a bit shocked that I’d came back, she was like, ‘why are you still here’, I was like ‘I’ve still got 6 hours left’ [laughs]. Erm, I think I’d wanted another job after that – I didn’t wanna end on that. Erm, there was definitely dark humour, which if you don’t laugh, you’ll cry. Which I think I definitely would if there wasn’t a couple of jokes here and there. (Participant 8)

I spoke to my mom, but I didn’t mention everything. I knew that she’d be sympathetic, and she’d care, but she wouldn’t understand. And I didn’t feel like I needed to put that on her when I knew it wouldn’t make that much difference. (Participant 8)

Some participants identified that ambulance staff utilised banter and dark humour as a specific means of coping with work-related stress. Acknowledging this, Participant 7 relayed that while such humour was acceptable among ambulance staff, she certainly would not share it with her family. Although finding it initially ‘harsh’, Participant 2 described why he later considered such humour beneficial:

Because it helps you sort of dissociate. When you come home you’re not bringing work home with you. If you thought about every sick patient that you’re taking in every week, you’d drive yourself crazy [laughs]. (Participant 2)

### The potential barrier of supernumerary status

Supernumerary status within the pre-hospital workplace was cited by some participants as an impediment to their inclusion. This was described by Participant 8 as feeling like a non-essential ‘extra’ and being ‘in the way’. This extended to attending calls and having to explain to service users why three clinicians had arrived on their doorstep:

We feel like we’re intruding on their normal day-to-day work . . . when you go to a patient’s house, they’re like, ‘why is there three of you?’. And then you have to introduce yourself as a university student, I think that kind of puts the barrier up between you and the other staff. (Participant 8)

Several participants relayed that ambulance staff would refer to them as ‘student’ rather than by their name. While Participant 1 said that he would not allow this to annoy him, he did infer that it was something which highlighted a difference between him and the crew:

It’s just sometimes you hear those little things and you sort of well . . . If they’re not bothering to learn my name to sign on sort of thing, you’re just called, ‘student’ . . . (Participant 1)

This perceived difference became more apparent through comparisons with another group of apprenticeship-style students who are employed by the local ambulance trust as emergency medical technicians. These students – colloquially known as ‘tech-to-paras’ owing to their progression towards paramedic status – differ in that they work as part of an ambulance crew and wear the same uniform. Some participants expressed that despite having similar abilities, ‘tech-to-paras’ were treated like members of staff, while supernumerary university students were generally held in lower regard:

I feel like, because they’ve got the uniform and they can drive, they get a lot more respect than we do. Yeah, even though we’re probably on the same level skills-wise and knowledge-wise, they still get that, that respect, ’cos they’re there for longer and they’re part of the crew. (Participant 7)

There’s this one guy that I can think of in particular, he kind of always [says], ‘make the tea, you’re the student’ – but he won’t speak like that to the tech-to-paras. (Participant 4)

### Benefitting from the informal support of student peers

Several participants recounted how they felt a sense of kinship with their student peers, and that this was a valuable support mechanism. As someone who admitted being generally quiet around ambulance staff, Participant 3 relayed that her university peers would enquire after each other following a difficult call, and that they would often ‘chill out’ together. She also stated that having once been reprimanded by her mentor it was only the support of her student peers that enabled her to continue:

It’s quite nice that we have that support, ’cos we’re all in the same situation at the end of the day . . . if I didn’t have them, I don’t think I would have gone into work and that sounds really bad. (Participant 3)

Other participants similarly found that talking to their peers represented a useful support mechanism and a means of discussing elements of their own mentorship with people who could understand:

Erm . . . it’s cool to say if you get like a job and you don’t agree with the mentor on it as much. It’s good to like relay back to like your friends at uni and see what they think, and if it lines up or how it differs . . . (Participant 6)

It did help to know there was someone else in the same situation. It was nice to have someone to talk to about it that understood. (Participant 7)

For some participants the uniqueness of the working environment and the nuances of the pervading culture made them feel unable to discuss many of their experiences with their family or non-ambulance friends. In this respect, student peers represented a body of people who could offer some understanding. Participant 5 makes his perception of this clear:

’Cos you can’t really talk about the jobs we go to to normal people . . . you just can’t. So, and if [students] are in the same cohort as you they’re your friends, they understand where you’re coming from, what level you’re at, and that seeing these things . . . (Participant 5)

### The ubiquitous nature of online chat groups

Almost all participants disclosed that they were part of a specific student-led online chat group and alluded to it as a means of support. The frequency and degree of participation among a cohort of approximately 100 students was conveyed by Participant 4:

We’ve got like a group as well on Facebook and it’s really handy. If you’re ever not sure of something . . . we all talk on it like once a day and send a message here and there. There’s probably only about 5 to 10 on the course that are not in it – so pretty much everyone’s in it. (Participant 4)

Participants described how operational experiences (appropriately devoid of patient details) would sometimes be shared within the chat group, and that it also represented a useful forum for posing course-related questions:

I’m part of all the WhatsApp groups that they’re in. I find that there’s a lot of erm, interaction with the students, if there’s information that I need, I will ask, and I’ll speak to people. (Participant 9)

I’ve actually gone to the group again for some of the stuff I don’t understand in the competency book, you know the book you have to sign off. I was really confused like – which part do we sign, so I asked them lot and they helped. (Participant 11)

You got any problems, you just put it into the chat . . . (Participant 12)

## Discussion

In alignment with prior research involving employed ambulance staff, the findings suggest that university paramedic students may benefit from informal support structures as a means of managing workplace-related stress. As can be seen, this includes involvement with the camaraderie and humour which is thought to represent an important means of diffusing tension after a traumatic incident (Clompus & Albarran, 2016; Jonsson & Segesten, 2003). While it has also been suggested that females may experience greater difficulty in engaging with the banter of an historically male-dominated culture (Clompus & Albarran, 2016), there was no suggestion within this study that gender was an impediment for the female participants.

While the literature relating to supernumerary student status within the pre-hospital workplace is limited, this expression of marginalisation does align with some of the older nursing research. Within an incident analysis involving student nurses, Joyce (1999) found that not being a part of the employed workforce led to supernumerary students experiencing negative attitudes and a degree of exclusion. In an interview study with qualified nurses, Hyde and Brady (2002) also found that unfavourable comparisons with employed ‘rostered’ nursing students (akin to ‘tech-to-para’ students) led to some workplace alienation. While there is some professional disparity with nursing research, the suggested potential for isolation may be important for the well-being of supernumerary paramedic students who may feel drawn towards the camaraderie of ambulance staff, but occupy a uniquely peripheral position within the workplace.

Being part of an informal student community may thus represent an important coping resource and an outlet for discussing concerns. The findings suggest that this may represent an outlet for things which they may feel unable to share with their mentor, and which they may feel family and non-ambulance friends would be unable to understand; a feeling succinctly encapsulated by Participant 5, as being unable to talk to ‘normal’ people. This is reflective of the compartmentalisation found to occur among qualified ambulance staff who avoid burdening loved ones with the details of traumatic incidents (Clompus & Albarran, 2016). However, while Shakespeare-Finch et al. (2002) suggest that for qualified staff this may be due to formal training and experience, this is less plausible for students with limited pre-hospital experience.

While the psychological benefits of peer support per se are explored within the literature (Green, 2018; Pinks et al., 2021; Rolfe et al., 2020), there is little involving paramedic students, or the potential immediacy and capacity for inclusion offered by contemporary online chat groups. Despite the professional dissonance, the potential value of this medium is suggested by Mercieca and Kelly (2018), who found that among newly qualified teachers, private social network sites represent a pre-dominant means for creating a supportive social community of peers. While paramedic students may use this medium in a similar manner, it may not be something which is readily accounted for by educators (and certainly not within the author’s experience). This may be especially so if – as with this study – the groups are created and managed by the students themselves.

### Recommendations for clinical practice and future research

Ambulance staff and educators should be aware of how potentially isolated supernumerary university paramedic students may feel when learning within the pre-hospital environment. Supernumerary status and feeling unable to talk to ‘normal’ people outside of the workplace may render them particularly vulnerable. Further research into how student paramedics manage their workplace stress is needed.

Informal online peer networks may represent an important means of support for supernumerary university paramedic students while on placement. Educators ideally need an awareness of how they may be implemented and used to ensure that they represent a supportive and inclusive space. Further research into this potentially valuable informal support structure is needed.

### Limitations

A key limitation is that these findings arose from an overarching phenomenological enquiry into the professional identity development of paramedic students. There was therefore no specific focus upon stress management or informal support structures. The purposive sampling approach has also been described as ‘highly prone to researcher bias’ (Sharma, 2017, p. 751), something which could be considered a limitation despite the author’s attempted reflexivity. This risk may even be amplified if, as with this study, the interpretation of data is undertaken by a lone researcher (Gregory, 2019). It is also quite possible that despite assurances of impartiality and anonymity, participant answers could have been tailored in accordance with their perceptions of the interview.

## Conclusions

Many of the supernumerary university paramedic students within this study seem to have accessed and drawn solace from the informal support of ambulance staff after a traumatic or upsetting experience. However, the findings also suggest that their supernumerary status may render them isolated from the pre-hospital workplace community. This is concerning when contextualised by suggestions that students may also compartmentalise traumatic experiences away from non-ambulance friends and loved ones.

The findings additionally suggest that online chat groups represent a ubiquitous medium for informal support among university paramedic students. Within this study, such groups were created and managed by the students themselves. If this is the norm, educators may not be aware of how this medium is implemented and used for informal support by student groups, nor can they ensure that all students have the same opportunity to be included.

## Author contributions

Mark Garratt was the sole researcher and author of the article. The findings are drawn from an overarching study undertaken for a doctoral research thesis at Staffordshire University (https://ethos.bl.uk/OrderDetails.do?did=1&uin=uk.bl.ethos.851733). MG acts as the guarantor for this article.

## Conflict of interest

None declared.

## Ethics

Approval was obtained from the Health Sciences Ethics Panel at Staffordshire University. Local ‘Gatekeeper’ permission and faculty ethical approval was also obtained from Coventry University (project reference no. P78111). Written informed consent was gained from each participant prior to interview.

## Funding

None.
